# Quantifying the metabolic-inflammatory axis: synergistic value of TyG index and FAI in assessing CAD risk among MAFLD patients

**DOI:** 10.3389/fendo.2026.1832661

**Published:** 2026-05-28

**Authors:** Xiaolong Bai, Zhijun Wang, Ziyi Cao, Junping Zhen

**Affiliations:** 1 College of Medical Imaging, Shanxi Medical University, Taiyuan, Shanxi, China; 2 Department of Imaging, Second Hospital of Shanxi Medical University, Taiyuan, Shanxi, China; 3 Second Clinical Medical College, Shanxi Medical University, Taiyuan, Shanxi, China; 4 First Clinical Medical College, Shanxi Medical University, Taiyuan, Shanxi, China; 5 Beijing Obstetrics and Gynecology Hospital, Capital Medical University, Beijing, China

**Keywords:** coronary functional ischemia, coronary heart disease, fat attenuation index, metabolic dysfunction-associated fatty liver disease, triglyceride-glucose

## Abstract

**Background:**

This study evaluated coronary inflammation and insulin resistance (IR) using pericoronary fat attenuation index (FAI) and triglyceride-glucose (TyG) index, and assessed their associations with coronary heart disease (CHD) and functional ischemia in patients with metabolic dysfunction-associated fatty liver disease (MAFLD).

**Methods:**

A total of 435 patients (174 MAFLD, 261 MAFLD+CHD) were included. The MAFLD+CHD group was stratified by CT-derived fractional flow reserve (CT-FFR) into >0.80 and ≤0.80 subgroups. FAI and CT-FFR were automatically calculated using AI-based software; Independent risk factors were identified using multivariable logistic regression analysis, based on which a nomogram was constructed. The predictive performance of the nomogram was evaluated using ROC curves, calibration curves, and decision curve analysis (DCA), with internal validation performed via the Bootstrap method. The predictive accuracy of the nomogram was also compared with that of FAI and the TyG index. Restricted cubic spline (RCS) models were employed to explore the potential nonlinear relationship between the TyG index and FAI, as well as the dose–response associations of these indices with disease risk.

**Results:**

Multivariate logistic regression showed that TyG index and FAI were independent risk factors for CHD and coronary functional ischemia (P<0.05), with CHD prevalence, functional ischemia, and TyG levels increasing alongside FAI. In MAFLD patients, the nomogram demonstrated excellent discrimination (ROC-AUC 0.952, 95% CI 0.935–0.970), and DCA, clinical net reduction, and clinical impact analyses confirmed its substantial clinical value in identifying high-risk CHD patients. For MAFLD patients with CHD, the nomogram also effectively predicted coronary functional ischemia (AUC 0.904, 95% CI 0.869–0.939). While FAI and TyG, alone or combined, had predictive value, the nomogram outperformed all single and combined indicators (AUCs 0.952 and 0.904; P<0.001). RCS analysis revealed a linear positive correlation be-tween TyG and FAI, both positively associated with CHD and functional ischemia risk.

**Conclusion:**

In patients with MAFLD and those with concomitant CHD, FAI was significantly positively correlated with the TyG index, and both were associated with the occurrence of CHD and coronary functional ischemia, suggesting that systemic metabolic dysfunction may promote disease progression through local coronary inflammation. Furthermore, a nomogram integrating the TyG index, FAI, and key clinical variables demonstrated excellent discriminative ability in internal validation, providing incremental diagnostic value for early CHD risk stratification in MAFLD patients.

## Introduction

Metabolic Dysfunction-Associated Fatty Liver Disease (MAFLD) has become a chronic liver disease that poses a global public health threat. Its clinical significance extends beyond the liver, as it is now recognized as a systemic disease affecting metabolism and the cardiovascular system (1). Epidemiological studies indicate that cardiovascular disease has overtaken liver-related complications as the leading cause of death in MAFLD patients (2). Although the association between MAFLD and coronary heart disease (CHD) has been well established, anatomical stenosis shown by coronary computed tomography angiography (CCTA) alone often fails to accurately reflect coronary functional status (3). Moreover, the commonly observed discrepancy between anatomical stenosis and functional ischemia in clinical practice suggests that hemodynamically significant ischemia is the key determinant of prognosis and guides clinical intervention (4). To address this clinical need, computed tomography angiography-derived fractional flow reserve (CT-FFR) provides a tool that bridges anatomical assessment and functional evaluation (5). As a non-invasive method based on computational fluid dynamics, CT-FFR shows good agreement with invasive FFR. Current studies indicate that a CT-FFR value ≤ 0.80 can serve as a critical threshold for identifying significant hemodynamic disturbances in the coronary arteries (6). However, despite its ability to noninvasively assess coronary hemodynamic abnormalities, the clinical application of CT-FFR is often limited by factors related to contrast agents. Therefore, the development of clinically accessible, cost-effective, and reliable noninvasive assessment indicators is of great practical significance for the early identification and intervention of coronary ischemia in patients with MAFLD. Current evidence suggests that coronary hemodynamic abnormalities are not solely determined by luminal stenosis; their underlying mechanisms also involve systemic metabolic dysregulation and low-grade chronic inflammation induced by hepatic steatosis, which together remodel the coronary vascular microenvironment (7, 8). However, in patients with MAFLD, whether systemic metabolic disturbances further mediate and accelerate the progression of local coronary inflammation remains to be quantified through reliable clinical indicators.

In recent years, pericoronary adipose tissue (PCAT) has attracted considerable attention due to its active endocrine and immune functions. Among its imaging markers, the fat attenuation index (FAI) serves as a non-invasive indicator of local coronary inflammation and can sensitively capture remodeling of the vascular microenvironment (9). Insulin resistance (IR) is now recognized as a central component of systemic metabolic dysfunction in MAFLD, and the triglyceride-glucose (TyG) index, a simple and reliable surrogate for IR, can indirectly reflect overall metabolic burden (10).

It remains unclear whether the TyG index, reflecting systemic metabolic load, is intrinsically associated with the FAI, which reflects local coronary inflammation, and whether this potential “metabolism–inflammation” interplay is related to the occurrence of CHD and coronary functional ischemia in patients with MAFLD. In particular, within the specific metabolic context of MAFLD, the relationship of the TyG index and FAI with CHD occurrence and CT-FFR–defined functional ischemia still lacks sufficient clinical evidence.

Based on this, the present study enrolled MAFLD patients with suspected CHD confirmed by abdominal ultrasonography, combining coronary angiography (CAG) to assess anatomical stenosis and CT-FFR to evaluate functional coronary ischemia. This study aims to explore the role of the TyG index as a bridge between systemic metabolic abnormalities and local coronary vascular microenvironment alterations, and to evaluate the association and clinical utility of the TyG index and FAI with coronary anatomical lesions and functional ischemia in patients with MAFLD. In addition, a nomogram model based on these relevant indicators was developed to provide a novel pathophysiological perspective and clinical basis for early risk identification and noninvasive functional assessment of cardiovascular disease in MAFLD patients.

## Materials and methods

### Study population

Patients with suspected CHD and MAFLD (confirmed by abdominal ultrasonography) who underwent CCTA at the Second Hospital of Shanxi Medical University between June 2019 and July 2025 were enrolled. Suspected CHD patients subsequently underwent CAG, and based on CAG results, patients were classified into two groups: MAFLD alone and MAFLD combined with CHD. For further stratified analysis, MAFLD+CHD patients were divided into CT-FFR > 0.80 and CT-FFR ≤ 0.80 subgroups using CCTA imaging data in combination with AI-based coronary analysis software.

This study strictly adhered to the principles of the Declaration of Helsinki and was approved by the Ethics Committee of the Second Hospital of Shanxi Medical University (approval number: 2025YX-183).

MAFLD was diagnosed based on the recommendations of the Asia Pacific Working Group, which primarily include the presence of fatty liver confirmed by imaging or histological methods. Alcohol consumption, a history of viral hepatitis, and the use of hepatotoxic drugs must be excluded. The presence of fatty liver was further confirmed by a standard liver ultrasound, performed by a trained operator, using enhanced liver echo contrast against the renal cortex as the diagnostic method for assessing liver steatosis (11). CHD diagnosis relied on CAG, performed using the Judkins technique. CHD was diagnosed when the diameter of any of the following vessels or their main branches was narrowed by 50% or more: Left Main Coronary Artery (LM), Left Anterior Descending artery (LAD), Left Circumflex artery (LCX), and Right Coronary Artery (RCA) (12). The exclusion criteria were as follows: patients with malignancies or a history of cardiac surgery; those with severe infections or congenital/structural heart disease; individuals with severe hepatic or renal dysfunction; patients who had recently used statins or other lipid-lowering drugs, hypoglycemic agents, or antihypertensive medications; and those with incomplete imaging data or failed CT-FFR analysis.

### Data collection and measurements

The study collected patients’ basic clinical data, including sex, age, history of hypertension, history of diabetes, smoking status, and body mass index (BMI). Laboratory measurements included total cholesterol (TC), triglycerides (TG), fasting blood glucose (FBG), high-density lipoprotein cholesterol (HDL-C), low-density lipoprotein cholesterol (LDL-C), creatinine (Scr), white blood cell count (WBC), neutrophil count (NEU), alanine aminotransferase (ALT), aspartate aminotransferase (AST), and the TyG index. Imaging indicators included the fat attenuation index (FAI) and CT-derived fractional flow reserve (CT-FFR). The calculation formula for the TyG index is as follows:


TyG index=ln(TG(mg/dL)×FBG(mg/dL)/2)

### CCTA imaging protocol

Dynamic volume scanning for CCTA was performed using the GE Revolution 256. The scanning range was set with the patient in a supine position, arms raised above the head, and the head advanced. The scan range extended from the tracheal bifurcation to 2 cm below the apex of the heart, with the lateral range covering the entire heart silhouette. The “smart monitoring method” was used for trigger control, with a threshold set at 120 HU. The scan delay time was 6 seconds, with the ascending aorta as the monitoring point threshold. The German Ulrich high-pressure injector was used to administer iodine contrast (350 mgI/ml) at a flow rate of 4.5–5 ml/s through the right elbow vein, with a dose of 60–80 ml, followed by an additional injection of 40–50 ml of saline. After the contrast agent injection, when the CT value at the monitoring point reached the set threshold, the patient was instructed to hold their breath at the end of inspiration, and the scan was started and continued until completion.

CCTA scanning parameters: Tube voltage 120 kV, tube current 300–600 mA (automatic adjustment), CCTA reconstruction thickness 0.625 mm, slice spacing 0.625 mm, FOV 250 mm × 250 mm, acquisition matrix size 512 × 512.

### Image postprocessing

**Figure 1:**
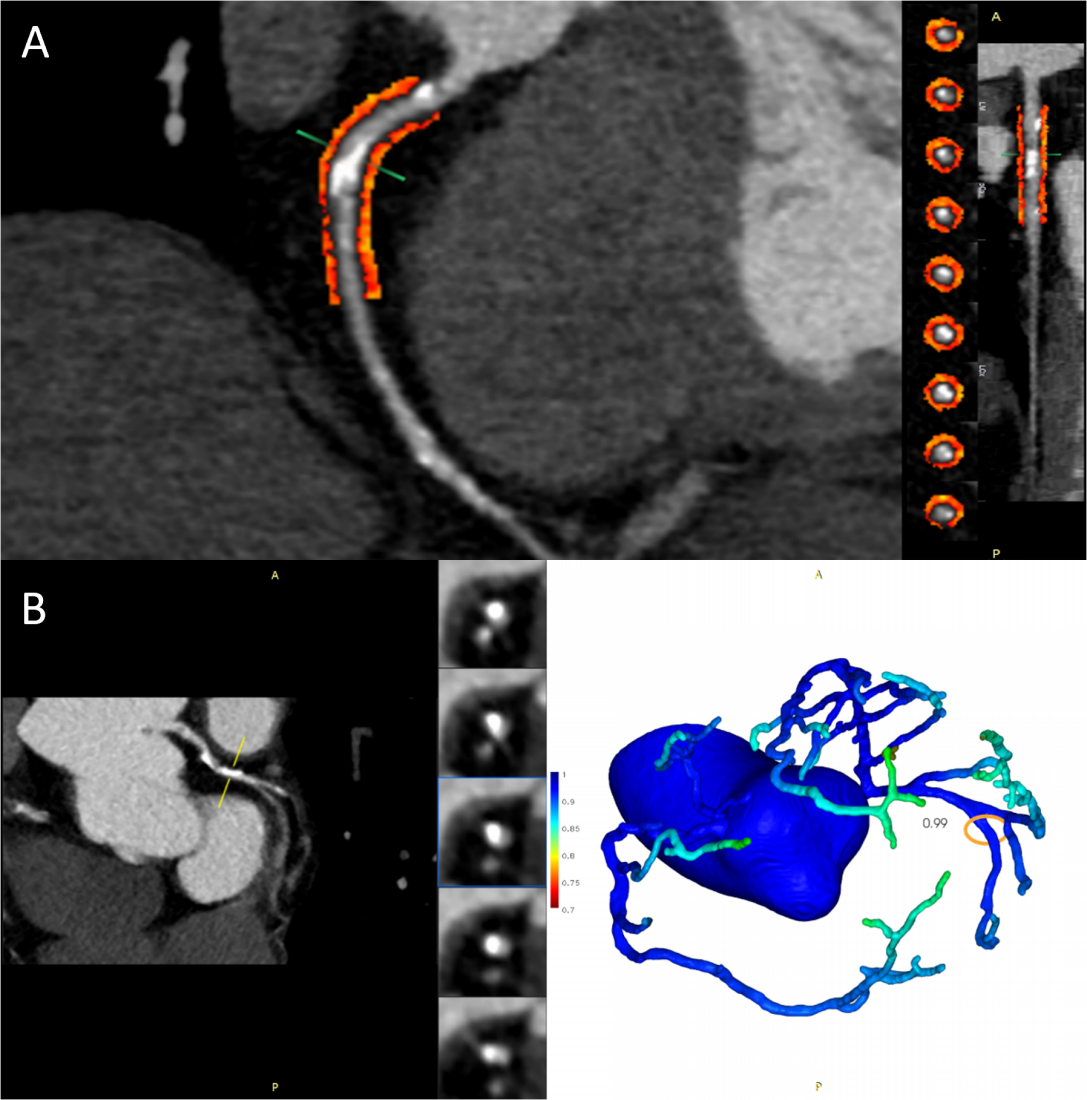
Imaging techniques used for the measurement of key cardiovascular parameters in MAFLD and CHD patients. **(A)** The computed tomography angiography surface plane reconstruction image, combined with fat segmentation, is used for the measurement of Fat Attenuation Index. **(B)** The computed tomography angiography surface plane reconstruction image and VR coronary artery tree color map are used for the measurement of CT fractional flow reserve values.

CCTA image data were uploaded to a GE AW4.7 workstation and processed using specialized coronary AI software. Systematic reconstruction was performed with post-processing and segmentation algorithms, including multi-planar reconstruction (MPR), volume rendering (VR), and curved planar reconstruction (CPR), to create a 3D coronary artery tree model. For FAI measurement, PCAT was defined as the adipose area within a radial distance equal to the vessel diameter from the outer vessel wall (13). FAI values, representing the mean CT attenuation of voxels within the PCAT (ranging from -190 HU to -30 HU), were automatically calculated to quantify coronary inflammation (14) (**Figure 1A**). Specifically, the software identified key segments: the proximal 40 mm of the LAD and LCX arteries, and the 10–50 mm proximal segment of the RCA, excluding any aortic influence. These fatty tissues were color-coded and displayed across MPR, CPR, and probe images, with the maximum FAI value among the three vessels selected as the representative patient-level value for risk assessment. Simultaneously, CT-FFR was used for hemodynamic evaluation based on computational fluid dynamics (15). Using the 3D coronary model, CT-FFR values were calculated across the arterial tree. For non-stenotic vessels, measurements were taken 2 mm from the vessel end; for stenotic vessels, measurements were made 2 cm distal to the most severe plaque, or 2 mm from the vessel diameter if the distal segment was too narrow. The minimum CT-FFR value among the three main branches was recorded for each patient, with a threshold of ≤ 0.80 indicating hemodynamically significant functional ischemia (16) (**Figure 1B**). All measurements were performed by two senior radiologists using a double-blind method, excluding any cases where manual diagnoses and AI predictions were inconsistent to ensure data reliability.

### Statistical analysis

Data were analyzed using SPSS 26.0 and R software. Continuous variables were first assessed for normality using the Kolmogorov–Smirnov test. Normally distributed data are presented as mean ± standard deviation (X̄ ± S) and compared between groups using an independent-samples t-test or one-way analysis of variance (ANOVA). Non-normally distributed data are expressed as median (M [Q1, Q3]) and compared using the Mann–Whitney U test or Kruskal–Wallis H test. Categorical variables are presented as counts and percentages [n (%)], with group differences assessed using the Chi-square test or Fisher’s exact test, as appropriate. Multicollinearity among variables was evaluated using the variance inflation factor (VIF).

Univariate and multivariate logistic regression analyses were conducted to identify factors associated with CHD in MAFLD patients and with CT-FFR ≤ 0.80 in MAFLD patients with CHD. Independent variables identified in the multivariate model were used to construct a nomogram. The performance of the nomogram was assessed comprehensively using multiple metrics. Discrimination was quantified by the area under the receiver operating characteristic curve (AUC-ROC) to evaluate its ability to distinguish CHD and coronary functional ischemia in MAFLD patients. Calibration was assessed using the Hosmer–Lemeshow goodness-of-fit test, Spiegelhalter’s Z test, and calibration curves, with internal validation performed via 1, 000 bootstrap resamples to correct for potential overfitting and evaluate agreement between predicted and observed outcomes. Clinical utility was further evaluated using decision curve analysis (DCA), the clinical net reduction curve (CNRC), and the clinical impact curve (CIC). Additionally, the DeLong test was applied to compare the discriminative performance of the nomogram with that of the FAI and TyG index. Finally, restricted cubic spline (RCS) models with four knots (5th, 35th, 65th, and 95th percentiles) were constructed to explore potential nonlinear relationships between the TyG index and FAI and to examine the dose–response associations of these indices with disease risk.

## Results

### Comparison of general data

A total of 435 patients were included in this study, comprising the MAFLD group (N = 174) and the CHD combined with MAFLD group (N = 261). Baseline characteristics, laboratory, and imaging parameters were compared between the two groups. Compared with the MAFLD-only group, the CHD + MAFLD group had a higher pro-portion of males, older age, and higher rates of smoking and diabetes, with all differences being statistically significant (P< 0.001). Laboratory results showed that WBC, NEU, ALT, AST, TG, FBG, Scr, and TyG levels were significantly higher, while HDL-C levels were significantly lower in the CHD + MAFLD group compared with the MAFLD group (P< 0.05). Imaging indicators demonstrated that FAI values were significantly higher in the CHD + MAFLD group than in the MAFLD group (P< 0.001). No significant differences were observed between the two groups in the prevalence of hypertension, BMI, TC, or LDL-C levels (P > 0.05) (**Supplementary Table 1**).

Among CHD + MAFLD patients, no significant differences in general demo-graphic characteristics were observed between the functional ischemia group (CT-FFR ≤ 0.80) and the non-ischemia group (CT-FFR > 0.80) (P > 0.05). Laboratory results showed that WBC, NEU, TG, FBG, and TyG levels were significantly higher, while HDL-C levels were significantly lower in the CT-FFR ≤ 0.80 group compared with the CT-FFR > 0.80 group (P< 0.05 for WBC, NEU, TG, FBG, TyG; P< 0.001 for HDL-C). No significant differences were observed in the remaining laboratory parameters between the two groups (P > 0.05). Imaging indicators demonstrated that FAI values were significantly higher in the CT-FFR ≤ 0.80 group than in the CT-FFR > 0.80 group (P< 0.001) (**Supplementary Table 2**).

### Univariate and multivariate analyses

In this study, the occurrence of CHD in MAFLD patients and the presence of coronary functional ischemia (CT-FFR ≤ 0.80) in MAFLD patients with CHD were used as dependent variables, while patients’ demographic characteristics, laboratory parameters, and FAI were used as independent variables. Univariate logistic regression analysis, multicollinearity assessment, and backward stepwise multivariate logistic regression analysis were performed sequentially to identify relevant risk factors and minimize confounding effects (variables with VIF > 10 were excluded due to high multicollinearity).

For CHD occurrence in MAFLD patients, univariate analysis showed that male sex, age, smoking history, diabetes history, WBC, NEU, ALT, AST, TG, FBG, Scr, HDL-C, TyG index, and FAI were all significantly associated with CHD (P< 0.05). Multivariate analysis further identified age, smoking history, WBC, ALT, AST, Scr, TyG index, and FAI as independent risk factors for CHD in MAFLD patients (P< 0.05) (**Table 1**). For CT-FFR ≤ 0.80 in MAFLD patients with CHD, univariate analysis indicated that WBC, NEU, TG, FBG, HDL-C, TyG index, and FAI were significantly associated with functional ischemia (P< 0.05). Multivariate analysis showed that WBC, NEU, TyG index, and FAI were independent risk factors for coronary functional ischemia in this population (P< 0.05), whereas HDL-C served as a protective factor (OR = 0.014, 95% CI: 0.002–0.125, P< 0.001) (**Table 2**).

**Table 1:** Logistic regression analysis of factors associated with coronary heart disease risk in MAFLD patients.

Variables	Univariate analysis		Multivariate analysis	
	OR (95%*CI)*	*P*	OR (95%*CI)*	*P*
Male(n%)	2.526 (1.697-3.760)	***<0.001***		
Age (years)	1.049 (1.027-1.072)	***<0.001***	1.054 (1.015-1.095)	***0.006***
Smoking (n%)	2.493 (1.667-3.730)	***<0.001***	3.543 (1.734-7.243)	***0.001***
Diabetes mellitus (n%)	2.896 (1.775-4.724)	***<0.001***		
Hypertension (n%)	1.333 (0.905-1.965)	0.146		
BMI(kg/m2)	1.048 (0.993-1.107)	0.090		
WBC(*10^9/L)	1.392 (1.254-1.545)	***<0.001***	1.356 (1.141-1.613)	***0.001***
NEU(*10^9/L)	1.290 (1.140-1.459)	***<0.001***		
ALT(U/L)	1.320 (1.244-1.399)	***<0.001***	1.389 (1.259-1.531)	***<0.001***
AST(U/L)	1.556 (1.416-1.710)	***<0.001***	1.597 (1.377-1.852)	***<0.001***
TC(mmol/L)	1.073 (0.912-1.262)	0.397		
TG(mmol/L)	1.211 (1.140-1.288)	***<0.001***		
FBG(mmol/L)	4.063 (2.969-5.561)	***<0.001***		
Scr(mmol/L)	1.018 (1.001-1.034)	***0.032***	1.034 (1.006-1.064)	***0.019***
HDL-C(mmol/L)	0.069 (0.018-0.263)	***<0.001***	0.109 (0.011-1.124)	0.063
LDL-C(mmol/L)	1.388 (0.515-3.743)	0.517		
FAI(HU)	1.087 (1.061-1.113)	***<0.001***	1.054 (1.017-1.092)	***0.004***
TyG	1.669 (1.494-1.864)	***<0.001***	1.536 (1.305-1.808)	***<0.001***

The bolded values indicate P < 0.05, suggesting that the differences between groups are statistically significant.

**Table 2:** Logistic regression analysis of risk factors associated with CT-FFR ≤ 0.80 in CHD + MAFLD patients.

Variables	Univariate analysis		Multivariate analysis	
	OR (95%*CI)*	*P*	OR (95%*CI)*	*P*
Male(n%)	1.276 (0.751-2.167)	0.367		
Age (years)	0.997 (0.971-1.023)	0.808		
Smoking (n%)	1.300 (0.799-2.117)	0.291		
Diabetes mellitus	1.243 (0.743-2.079)	0.407		
Hypertension (n%)	1.339 (0.822-2.178)	0.241		
BMI(kg/m^2^)	1.031 (0.958-1.109)	0.414		
WBC(*10^9/L)	1.319 (1.163-1.495)	***<0.001***	1.522 (1.264-1.834)	***<0.001***
NEU(*10^9/L)	1.342 (1.121-1.605)	***0.001***	1.491 (1.150-1.932)	***<0.01***
ALT(U/L)	1.028 (0.977-1.082)	0.291		
AST(U/L)	1.041 (0.960-1.129)	0.334		
TC(mmol/L)	1.134 (0.930-1.384)	0.215		
TG(mmol/L)	1.147 (1.042-1.263)	***0.005***		
FBG(mmol/L)	1.113 (1.074-1.152)	***<0.001***		
Scr(mmol/L)	1.009 (0.989-1.029)	0.384		
HDL-C(mmol/L)	0.014 (0.003-0.073)	***<0.001***	0.014 (0.002-0.125)	***<0.001***
LDL-C(mmol/L)	1.802 (0.521-6.228)	0.352		
FAI(HU)	1.168 (1.119-1.220)	***<0.001***	1.205 (1.137-1.278)	***<0.001***
TyG	1.630 (1.384-1.920)	***<0.001***	1.644 (1.331-2.030)	***<0.001***

The bolded values indicate P < 0.05, suggesting that the differences between groups are statistically significant.

### Comparison of general data among patients with different FAI levels

To investigate the relationship between different FAI levels and the occurrence of CHD in MAFLD patients, the patients were divided into three groups (T1, T2, and T3; n = 145 per group) according to FAI tertiles (**Supplementary Figure 1A**). Comparison of baseline characteristics among the groups showed that sex, smoking rate, NEU, ALT, AST, TG, FBG, HDL-C, TyG, FAI, and CHD prevalence differed significantly across the three groups (P< 0.05). The prevalence of CHD increased significantly with higher FAI tertiles, and the TyG index also increased progressively with rising FAI levels (P< 0.05) (**Supplementary Figure 1B**; **Table 3**).

**Table 3:** Comparison of general characteristics among the three FAI groups in MAFLD patients.

Variables	T1 (n=145)	T2 (n=145)	T3 (n=145)	p
Male(n%)	76 (52.4%)	90 (62.1%)	99 (68.3%)	***<0.05***
Age (years)	55.32 ± 10.44	55.91 ± 8.84	56.21 ± 9.83	0.731
Smoking (n%)	49 (33.8%)	71 (49.0%)	72 (49.7%)	***<0.05***
Diabetes mellitus	31 (21.4%)	42 (29.0%)	41 (28.3%)	0.267
Hypertension (n%)	60 (41.4%)	69 (47.6%)	67 (46.2%)	0.537
BMI(kg/m^2^)	25.82 ± 3.65	26.46 ± 3.27	26.53 ± 3.69	0.176
WBC(*10^9/L)	6.59 ± 2.00	6.95 ± 2.16	7.05 ± 2.21	0.16
NEU(*10^9/L)	4.28 ± 1.70	4.82 ± 1.65	5.01 ± 1.55	***<0.05***
ALT(U/L)	24.93[22.09;28.77]	26.69[23.16;31.94]	26.71[23.46;29.80]	***<0.05***
AST(U/L)	20.99[19.02;22.96]	22.60[20.37;25.23]	23.09[20.46;25.38]	***<0.05***
TC(mmol/L)	4.64 ± 1.12	4.56 ± 1.22	4.65 ± 1.23	0.786
TG(mmol/L)	1.89[1.62;2.11]	1.93[1.74;2.22]	1.98[1.83;2.20]	***<0.05***
FBG(mmol/L)	5.75[5.28;6.24]	5.87[5.35;6.56]	6.23[5.56;7.01]	***<0.05***
Scr(mmol/L)	68.89 ± 12.80	67.81 ± 11.35	69.40 ± 12.36	0.525
HDL-C(mmol/L)	1.20[1.09;1.31]	1.21[1.09;1.31]	1.14[1.03;1.27]	***<0.05***
LDL-C(mmol/L)	2.51[2.38;2.69]	2.49[2.37;2.68]	2.51[2.38;2.61]	0.837
FAI(HU)	-87.06[-91.58;-83.94]	-78.40[-80.51;-76.08]	-68.27[-72.22;-65.28]	***<0.05***
TyG	9.08[8.86;9.21]	9.15[8.95;9.28]	9.21[9.05;9.34]	***<0.05***
CHD(n%)	56 (38.6%)	93 (64.1%)	112 (77.2%)	***<0.001***

The bolded values indicate P < 0.05, suggesting that the differences between groups are statistically significant.

To further explore the relationship between FAI gradient levels and the risk of coronary functional ischemia in MAFLD patients with CHD, these patients were di-vided into three groups (F1, F2, and F3; n = 87 each) according to FAI tertiles (**Supplementary Figure 2A**). Comparison among the groups showed that NEU, ALT, FBG, HDL−C, FAI, TyG, and the incidence of CT−FFR ≤ 0.80 differed significantly across the three groups (all P< 0.05). Moreover, with increasing FAI tertiles, the risk of CT−FFR ≤ 0.80 increased significantly, and the TyG index rose synchronously with FAI levels (P< 0.05) (**Supplementary Figure 2B**; **Table 4**).

**Table 4:** Comparison of general characteristics among the three FAI groups in MAFLD with CHD patients.

Variables	F1 (n=87)	F2 (n=87)	F3 (n=87)	p
Male(n%)	56 (64.37%)	63 (72.41%)	63 (72.41%)	0.411
Age (years)	57.44 ± 9.32	57.90 ± 8.71	57.26 ± 9.98	0.899
Smoking (n%)	42 (48.28%)	51 (58.62%)	45 (51.72%)	0.380
Diabetes mellitus	32 (36.78%)	29 (33.33%)	27 (31.03%)	0.722
Hypertension (n%)	40 (45.98%)	46 (52.87%)	39 (44.83%)	0.517
BMI(kg/m^2^)	26.43 ± 3.17	26.29 ± 3.33	26.79 ± 3.53	0.601
WBC(*10^9/L)	7.44 ± 2.08	7.39 ± 2.06	7.39 ± 2.36	0.979
NEU(*10^9/L)	4.46[3.72;5.60]	4.86[4.08;6.14]	4.99[4.22;6.15]	***0.043***
ALT(U/L)	28.87[25.38;32.05]	30.06[25.41;33.13]	27.85[24.12;30.58]	***0.033***
AST(U/L)	22.88[21.06;25.02]	23.00[21.61;26.03]	24.11[21.72;26.37]	0.112
TC(mmol/L)	4.62 ± 1.18	4.66 ± 1.28	4.69 ± 1.25	0.931
TG(mmol/L)	1.97[1.83;2.17]	1.97[1.81;2.19]	2.05[1.88;2.26]	0.254
FBG(mmol/L)	6.01[5.66;6.58]	6.36[5.76;7.17]	6.59[5.79;7.22]	***<0.05***
Scr(mmol/L)	69.49 ± 13.55	69.75 ± 11.37	69.93 ± 12.67	0.973
HDL-C(mmol/L)	1.13[1.03;1.29]	1.20[1.07;1.29]	1.09[0.96;1.25]	***0.018***
LDL-C(mmol/L)	2.52[2.39;2.72]	2.49[2.37;2.64]	2.52[2.39;2.64]	0.320
FAI(HU)	-82.59[-86.41;-81.52]	-75.47[-77.66;-73.67]	-66.88[-69.29;-64.23]	***<0.05***
TyG	9.17[9.07;9.28]	9.21[9.10;9.38]	9.29[9.17;9.37]	***<0.05***
CT−FFR ≤0.80 (n%)	13 (14.94%)	50 (57.47%)	67 (77.01%)	***<0.001***

The bolded values indicate P < 0.05, suggesting that the differences between groups are statistically significant.

### Construction of the nomogram model

**Figure 2:**
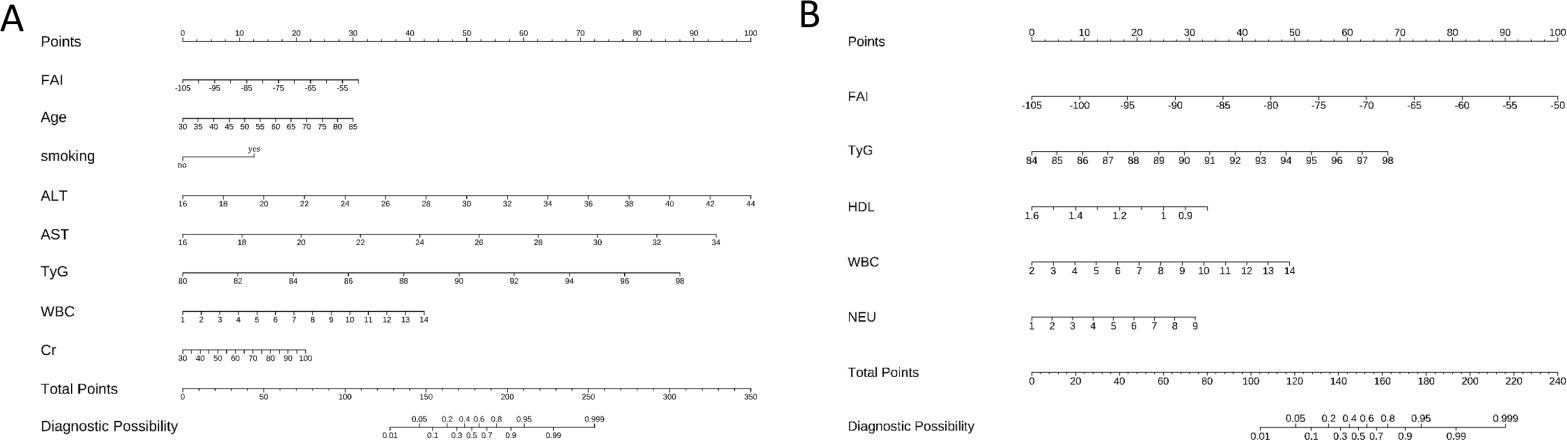
Nomograms for predicting the risk of CHD and functional ischemia in MAFLD patients **(A)** Nomogram model for predicting the risk of CHD in MAFLD patients **(B)** Nomogram model for predicting the risk of coronary functional ischemia in MAFLD patients with CHD.

In multivariate logistic regression analysis of MAFLD patients, age, smoking, WBC, ALT, AST, Scr, FAI, and TyG were identified as independent predictors of CHD. Based on these variables, a nomogram for predicting the risk of CHD in MAFLD patients was constructed. By summing the scores corresponding to each fac-tor to generate a total score and mapping it to the scale below, the probability of CHD occurrence for each patient can be estimated. A higher total score indicates a greater risk of CHD (**Figure 2A**). The performance of the nomogram was comprehensively validated, demonstrating excellent discriminative ability with an AUC of 0.952 (95% CI: 0.935–0.970). At the optimal cutoff determined by the maximum Youden index, the model achieved a sensitivity of 85.4% and a specificity of 92.5% (**Supplementary Figure 3A**). Internal validation using 1, 000 bootstrap resamples yielded an AUC of 0.955 (95% CI: 0.938–0.972), indicating good model robustness (**Supplementary Figure 3B**). Calibration was excellent, as shown by the Hosmer–Lemeshow goodness-of-fit test (X² = 4.564, P = 0.803), Spiegelhalter Z test (P = 0.958), and calibration curves, confirming close agreement between predicted probabilities and observed outcomes (**Supplementary Figure 4A**). Furthermore, DCA, CNRC, and CIC indicated that the nomogram provided substantial net clinical benefit (**Supplementary Figures 4B–D**). Compared with traditional assessment methods, this predictive model offers superior clinical utility in identifying MAFLD patients at high risk of CHD.

In multivariate logistic regression analysis of MAFLD patients with CHD, WBC, NEU, HDL, FAI, and TyG were identified as independent predictors of coronary functional ischemia, with HDL serving as a protective factor. Based on these variables, a nomogram was constructed to predict the risk of coronary functional ischemia in this patient population (**Figure 2B**). Comprehensive validation of the nomogram demonstrated excellent discriminative performance, with an AUC of 0.904 (95% CI: 0.869–0.939). At the optimal cutoff determined by the maximum Youden index, the model achieved a sensitivity of 90.0% and a specificity of 73.3% (**Supplementary Figure 5A**). Internal validation using 1, 000 bootstrap resamples yielded an AUC of 0.905 (95% CI: 0.869–0.940), indicating robust model stability (**Supplementary Figure 5B**). Calibration was excellent, as evidenced by the Hosmer–Lemeshow goodness-of-fit test (X² = 3.143, P = 0.925), Spiegelhalter Z test (P = 0.901), and calibration curves, all showing close agreement between the apparent and bias-corrected curves with the ideal prediction line, confirming high model calibration (**Supplementary Figure 6A**). Furthermore, DCA, CNRC, and CIC indicated that the nomogram provides substantial net clinical benefit (**Supplementary Figures 6B–D**). Compared with traditional assessment methods, this predictive model demonstrates superior clinical utility for identifying MAFLD patients with CHD who are at high risk of coronary functional ischemia.

### Comparison of diagnostic value of TyG index, FAI, their combination and nomogram for coronary events in MAFLD patients

This study evaluated the diagnostic performance and clinical net benefit of individual indicators and nomogram models for predicting CHD and coronary functional ischemia in MAFLD patients using ROC curves and DCA. For predicting CHD, ROC analysis indicated that both the TyG index and FAI had moderate predictive value, with AUCs of 0.789 (95% CI: 0.745–0.832) and 0.706 (95% CI: 0.655–0.756), respectively. Combining the two indices improved predictive performance, yielding an AUC of 0.821 (95% CI: 0.781–0.860). The nomogram constructed from multivariate logistic regression exhibited the best performance, with an AUC of 0.952 (95% CI: 0.935–0.970), significantly outperforming TyG, FAI, and their combination (P< 0.001) (**Table 5**) (**Supplementary Figure 7A**). DCA further confirmed that the nomogram provided superior clinical net benefit (**Supplementary Figure 7B**).

**Table 5:** Table of ROC curve analysis for TyG index, FAI, their combination and nomogram model in predicting CHD risk in patients with MAFLD (P-values indicate the statistical differences in predictive performance among the TyG index, FAI, their combination, and the nomogram model).

Variables	AUC	95%CI	P	Sensitivity	Specificity	Optimal cutoff value
FAI	0.706	0.655-0.756	***<0.001***	0.659	0.678	-78.99
TyG	0.789	0.745-0.832	***<0.001***	0.824	0.626	9.045
FAI+TyG	0.821	0.781-0.860	***<0.001***	0.870	0.632	–
Nomogram	0.952	0.935-0.970		0.854	0.925	–

The bolded values indicate P < 0.05, suggesting that the differences between groups are statistically significant.

Similarly, for predicting coronary functional ischemia in MAFLD patients with CHD, both TyG and FAI demonstrated effective predictive ability, with AUCs of 0.726 (95% CI: 0.665–0.787) and 0.799 (95% CI: 0.744–0.853), respectively. When combined, the predictive performance improved further, reaching an AUC of 0.844 (95% CI: 0.798–0.891). The nomogram based on multivariate logistic regression showed the highest predictive ability, with an AUC of 0.904 (95% CI: 0.869–0.939), significantly higher than that of the individual indicators and their combination (P< 0.001) (**Table 6**) (**Supplementary Figure 8A**). DCA also indicated that the nomogram offered greater clinical net benefit (**Supplementary Figure 8B**).

**Table 6:** Table of ROC Curve Analysis of TyG Index, FAI, Their Combination and Nomogram Model for Coronary Functional Ischemia in Patients with Both CHD and MAFLD (P-values represent the statistical differences in predictive efficacy among the TyG index, FAI, their combination, and the nomogram model).

Variables	AUC	95%CI	P	Sensitivity	Specificity	Optimal cutoff value
FAI	0.799	0.744-0.853	***<0.001***	0.785	0.733	-76.035
TyG	0.726	0.665-0.787	***<0.001***	0.638	0.725	9.235
FAI+TyG	0.844	0.798-0.891	***<0.001***	0.785	0.802	–
Nomogram	0.904	0.869-0.939		0.900	0.733	–

The bolded values indicate P < 0.05, suggesting that the differences between groups are statistically significant.

### Linear dose–response of TyG and FAI on CHD risk and coronary dysfunction in MAFLD

**Figure 3:**
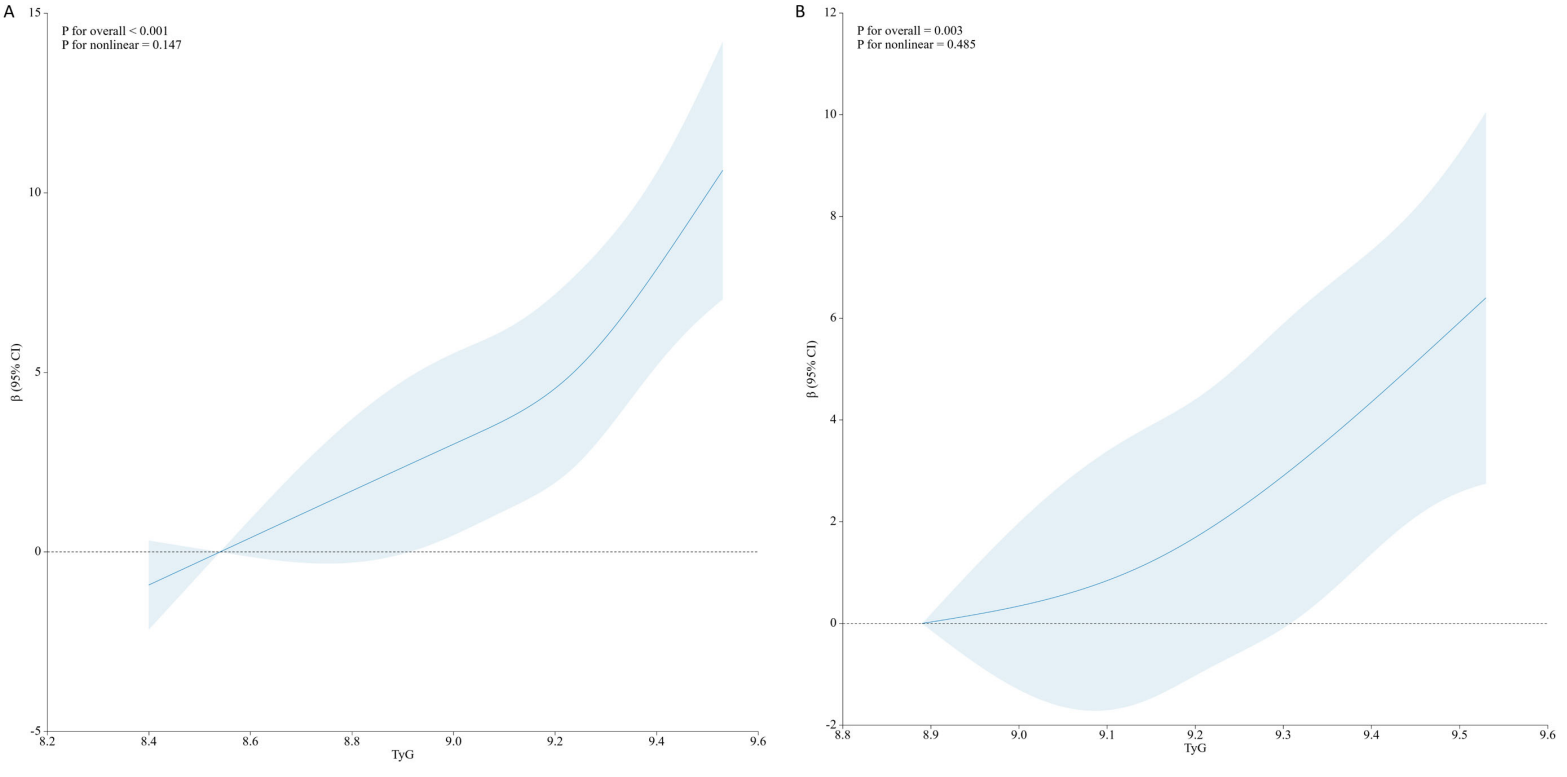
RCS curves showing the association between TyG index and pericoronary FAI in the MAFLD population **(A)** and in MAFLD patients with CHD **(B)**. The Y-axis represents the regression coefficient β (95% CI) of FAI, reflecting the quantitative change of FAI with varying TyG index. The solid line indicates the fitted β trend, and the shaded area represents the 95% confidence interval. The model was fitted using 4 knots (at the 5th, 35th, 65th, and 95th percentiles).

Univariate RCS regression analyses were performed in the overall MAFLD population and in the MAFLD with CHD subgroup to evaluate the effect of the TyG index on FAI levels. The results showed a significant linear dose–response relationship between the TyG index and FAI in the MAFLD population (P _overall_< 0.001, P _nonlinearity_ = 0.147), indicating a continuous linear increase in FAI with rising TyG index (**Figure 3A**). This linear association persisted in MAFLD patients with CHD (P _overall_ = 0.003, P _nonlinearity_ = 0.485) (**Figure 3B**).

**Figure 4:**
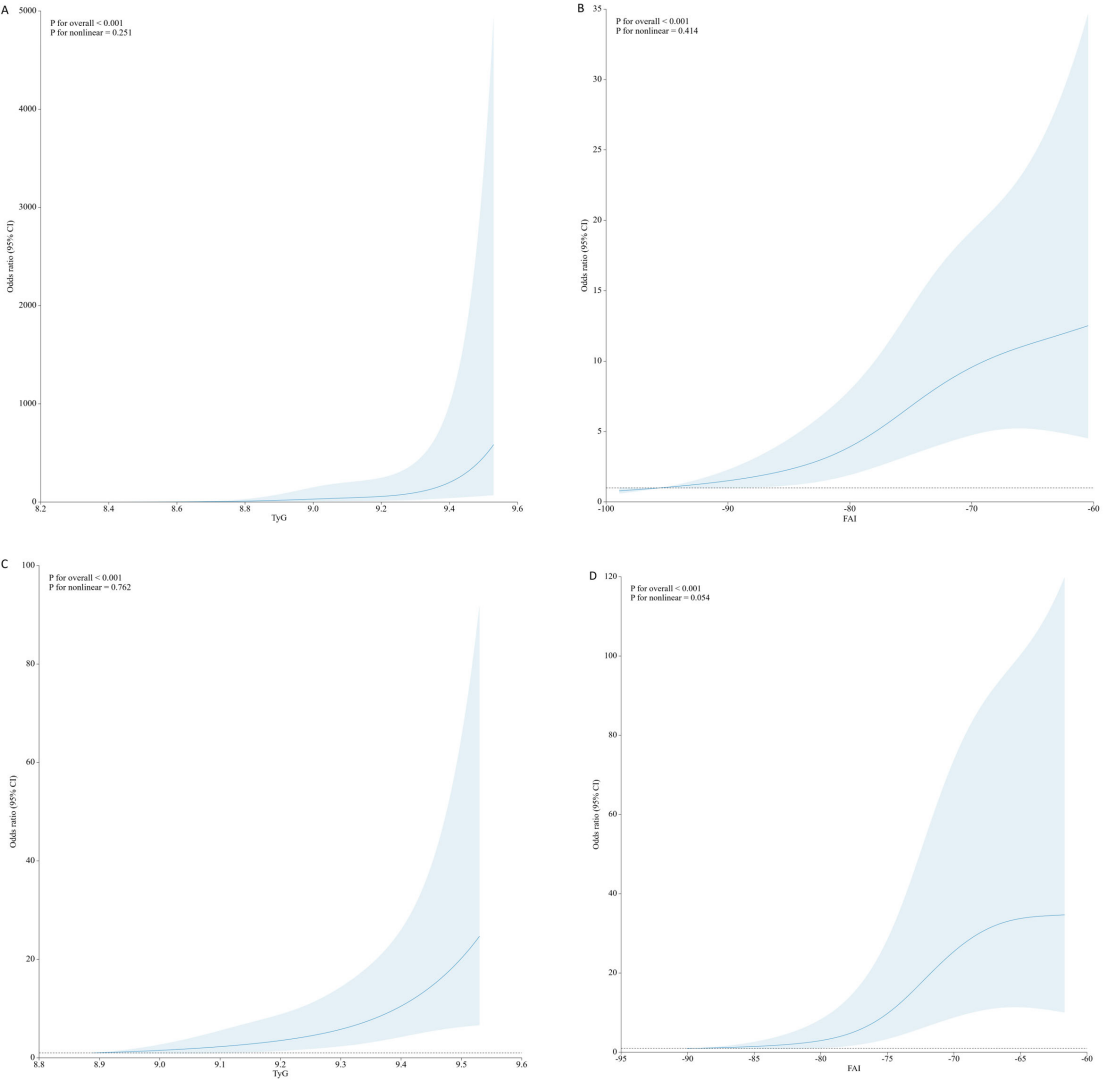
Association curves of TyG index and FAI with CHD risk and coronary functional ischemia risk in MAFLD patients, fitted using RCS. **(A)** Effect of TyG index on CHD risk in MAFLD patients. **(B)** Effect of pericoronary FAI on CHD risk in MAFLD patients. **(C)** Effect of TyG index on the risk of coronary functional ischemia in MAFLD patients with CHD. **(D)** Effect of pericoronary FAI on the risk of coronary functional ischemia in MAFLD patients with CHD. The Y-axis represents the odds ratio (OR) for CHD and functional ischemia risk. The solid line indicates the fitted OR curve, and the shaded area represents the 95% confidence interval (95% CI). The model was fitted using 4 knots based on the 5th, 35th, 65th, and 95th percentiles.

Further RCS model analyses were conducted to assess the dose–response relationships of the TyG index and FAI with the risk of CHD occurrence and coronary functional ischemia. The results demonstrated that both the TyG index and FAI were significantly positively associated with CHD risk in MAFLD patients, indicating that the risk of CHD (OR) increased continuously with higher levels of metabolic dysregulation and local inflammation (TyG, P _overall_< 0.001, P _nonlinearity_ = 0.251) (FAI, P _overall_< 0.001, P _nonlinearity_ = 0.414) (**Figures 4A, B**). In MAFLD patients with CHD, the TyG index and FAI were also significantly positively associated with the risk of coronary functional ischemia, suggesting that worsening insulin resistance and pericoronary inflammation corresponded linearly to an increased risk of impaired coronary perfusion(TyG, P _overall_< 0.001, P _nonlinearity_ = 0.762) (FAI, P _overall_< 0.001, P _nonlinearity_ = 0.054) (**Figures 4C, D**).

## Discussion

The rising prevalence of MAFLD has made its intrinsic association with CHD a major research focus in the fields of metabolic and cardiovascular medicine. It is now widely recognized that MAFLD is not limited to local pathological changes in the liver, but represents a systemic disease characterized primarily by whole-body metabolic dysregulation. Its impact on the cardiovascular system is not isolated, but is transmitted through multiple pathological pathways and targets, ultimately leading to CHD and coronary functional ischemia (17). Currently, the central role of IR–mediated glucose and lipid metabolic disorders is well established; however, whether these systemic metabolic abnormalities can be translated into local coronary vascular inflammation and functional impairment remains unclear, and precise quantitative indicators are lacking. Therefore, further investigation into the mechanisms by which MAFLD contributes to CHD and coronary functional ischemia is crucial for providing a theoretical basis for early intervention in high-risk cardiovascular populations.

IR, as a central component of systemic metabolic dysregulation in MAFLD, serves as a critical link between MAFLD and CHD. The metabolic disturbances mediated by IR not only initiate local hepatic pathology but also act as a key driver of cardiovascular injury (18). The TyG index, a simple and reliable surrogate marker of IR, can quantify systemic glucose and lipid metabolic burden, thereby indirectly reflecting the severity of IR and providing a practical tool for investigating the metabolic mechanisms underlying cardiovascular risk in MAFLD patients. From a pathophysiological perspective, abnormal hepatic lipid metabolism in MAFLD patients acts as the initiating factor for IR. Enhanced hepatic fatty acid uptake and synthesis, together with excessive lipid production that cannot be efficiently metabolized, lead to abnormal lipid accumulation within hepatocytes. This is accompanied by impaired glucose production regulation, resulting in concurrent local accumulation of both lipids and glucose, which drives the onset and progression of IR (19). Persistent IR, in turn, further exacerbates systemic glucose and lipid metabolic dysregulation, forming a vicious cycle of hepatic lipid accumulation–IR–systemic metabolic disturbance.

MAFLD is essentially a chronic low-grade inflammatory condition, characterized by elevated circulating inflammatory cytokines and sustained activation of pro-inflammatory signaling pathways. The propagation of this systemic inflammation to the local coronary environment represents a key intermediate step in the development of CHD and coronary functional ischemia in MAFLD, and its efficiency largely depends on the remodeling effects of ectopic fat deposition on the vascular microenvironment, with pathological changes in PCAT playing a central mediating role (20). Traditionally, adipose tissue was considered merely an energy storage depot; however, modern metabolic research has demonstrated that in the context of MAFLD, hepatic lipid accumulation triggers local inflammation, activating immune cells that release pro-inflammatory factors such as CCL2 and MCP1. These inflammatory mediators enter the circulation and act on PCAT, inducing aberrant adipocyte differentiation, macrophage infiltration, and collagen deposition, ultimately leading to PCAT inflammatory remodeling (21). PCAT, as a metabolically active endocrine and immunoregulatory tissue, interacts closely with adjacent coronary vessel walls through paracrine signaling or “outside-in” transduction mechanisms, thereby regulating vascular structure and function. Its pathological alterations directly contribute to the initiation and progression of atherosclerosis. The FAI, a novel non-invasive imaging marker, can quantify the degree of PCAT attenuation, accurately reflecting the inflammatory status and structural remodeling of PCAT (22). Its core value lies in converting local inflammatory processes into quantifiable imaging indicators, providing an intuitive means to assess the translation of systemic inflammation into localized vascular inflammation.

IR can directly impair vascular endothelial function and induce inflammation through oxidative stress, thereby leading to coronary artery dysfunction. In addition, IR can reduce nitric oxide production, increase vascular stiffness, and promote a hypercoagulable state, disrupting the balance between bleeding and coagulation and increasing the risk of thromboembolic events, which in turn accelerates PCAT inflammation remodeling. Consequently, under IR conditions, endothelial cells may exhibit heightened sensitivity to pro-inflammatory stimuli. Previous studies have demonstrated that the chronic low-grade systemic inflammation observed in MAFLD patients differs from acute infection-induced inflammation, being long-lasting and low-intensity, and it can manifest even in the early stages of impaired insulin secretion, showing a close interrelationship with IR (23). This association is particularly pronounced in obesity-related MAFLD. Obesity-induced remodeling of visceral adipose tissue promotes macrophage activation and migration into adipose tissue, leading to the release of pro-inflammatory mediators such as interleukins and tumor necrosis fac-tor, creating a pro-inflammatory microenvironment and impairing insulin signaling in adipocytes, thereby further aggravating IR (24). In turn, IR promotes macrophage activation, establishing a vicious cycle of systemic metabolic dysregulation–systemic inflammation–PCAT remodeling–local vascular inflammation. This mechanism provides a potential pathological basis for the combined role of the TyG index and FAI in driving coronary artery injury. This view is supported by Tarantino et al., who reported a significant association between MAFLD and carotid intima–media thickness (IMT), a reliable surrogate marker of subclinical atherosclerosis reflecting the systemic metabolic–inflammatory burden (8). Moreover, persistent low-grade inflammation and lipotoxicity in MAFLD patients contribute to endothelial dysfunction and structural arterial changes, manifested as increased IMT. In the present study, by integrating the TyG index, which reflects systemic metabolic derangement, with FAI, which quantifies local coronary adipose remodeling, clinicians can more comprehensively assess the cumulative impact of insulin resistance and local inflammation on the vascular wall. This combined approach enables more precise vascular risk stratification in MAFLD patients and further reinforces the clinical relevance of our findings.

The results of this study demonstrated that in both MAFLD and MAFLD with CHD populations, FAI and the TyG index were significantly positively correlated. Moreover, both the TyG index and FAI showed significant positive associations with the presence of CHD and coronary functional ischemia. These findings support our hypothesis that elevated TyG index, reflecting insulin resistance and systemic glucose–lipid metabolic dysregulation, may lead to excessive release of free fatty acids, activating pro-inflammatory signaling pathways within PCAT. This, in turn, induces a reduction in adipocyte volume and an increase in interstitial water content, which is directly reflected in imaging as a local increase in FAI. In this context, changes in the TyG index may directly mirror the degree of inflammatory remodeling in PCAT, serving as a critical link between systemic metabolic disturbances and coronary functional impairment. During this process, insulin resistance and inflammation may form a vicious cycle, mutually reinforcing each other and exacerbating pathological progression. This dual-assessment approach highlights that the progression from MAFLD to vascular functional alterations is not coincidental, but rather driven by the convergence of metabolic stress and inflammatory signaling, thereby enhancing the ability to predict coronary heart disease and functional ischemia in MAFLD patients. Furthermore, the nomogram model constructed in this study, integrating the TyG index, FAI, and key clinical indicators, demonstrated excellent discriminative ability and internal validation performance in relation to CHD and coronary functional ischemia in MAFLD patients. This model offers the potential to provide a comprehensive assessment of the full pathway from systemic metabolic dysregulation to local vascular inflammation and ultimately to functional ischemia, thereby delivering incremental diagnostic value for early risk stratification of CHD in MAFLD patients.

Nevertheless, several limitations of this study should be acknowledged. First, the single-center retrospective design limited the sample size and generalizability, which may introduce selection bias. Second, this study used a CT-FFR value ≤ 0.80 as the threshold for defining functional ischemia. Although CT-FFR can effectively reflect coronary hemodynamic abnormalities, it was not validated against invasive FFR or clinical ischemic events in this cohort, while invasive FFR remains the gold standard for diagnosing coronary functional ischemia. No patients in this study underwent invasive FFR assessment, which may lead to potential false-positive or false-negative results when relying solely on CT-FFR, thereby affecting the accuracy of functional ischemia evaluation. Third, although patients with recent use of statins or other lipid-lowering drugs, glucose-lowering agents, and antihypertensive medications were excluded, residual confounding factors may still exist, such as long-term medication history, diet, physical activity, and other metabolic indicators, which could influence the TyG index and FAI and potentially bias the results. Future studies should be conducted in multicenter, large-sample, prospective cohorts, and incorporate multi-omics analyses (e.g., metabolomics, proteomics, single-cell immune profiling) to investigate the molecular mechanisms linking insulin resistance and local vascular microenvironment remodeling, thereby elucidating the full disease pathway of MAFLD-associated coronary functional impairment and providing a more precise basis for early intervention.

## Conclusion

In patients with MAFLD and CHD, FAI was significantly positively correlated with the TyG index, and both were associated with the presence of CHD and coronary functional ischemia, suggesting that systemic metabolic dysregulation may promote disease progression via local coronary inflammation. Furthermore, a nomogram integrating the TyG index, FAI, and key clinical indicators demonstrated good discriminative performance in internal validation, providing incremental diagnostic value for early CHD risk stratification in MAFLD patients.

## Data Availability

The raw data supporting the conclusions of this article will be made available by the authors, without undue reservation.
