# Supplementary material for: Quantifying the metabolic-inflammatory axis: synergistic value of TyG index and FAI in assessing CAD risk among MAFLD patients
Source: Front Endocrinol (Lausanne). 2026 May 28;17:1832661. doi: 10.3389/fendo.2026.1832661 (PMC13253315; doi:10.3389/fendo.2026.1832661)
Supplement: Supplementary file 1 [file DataSheet1.docx]

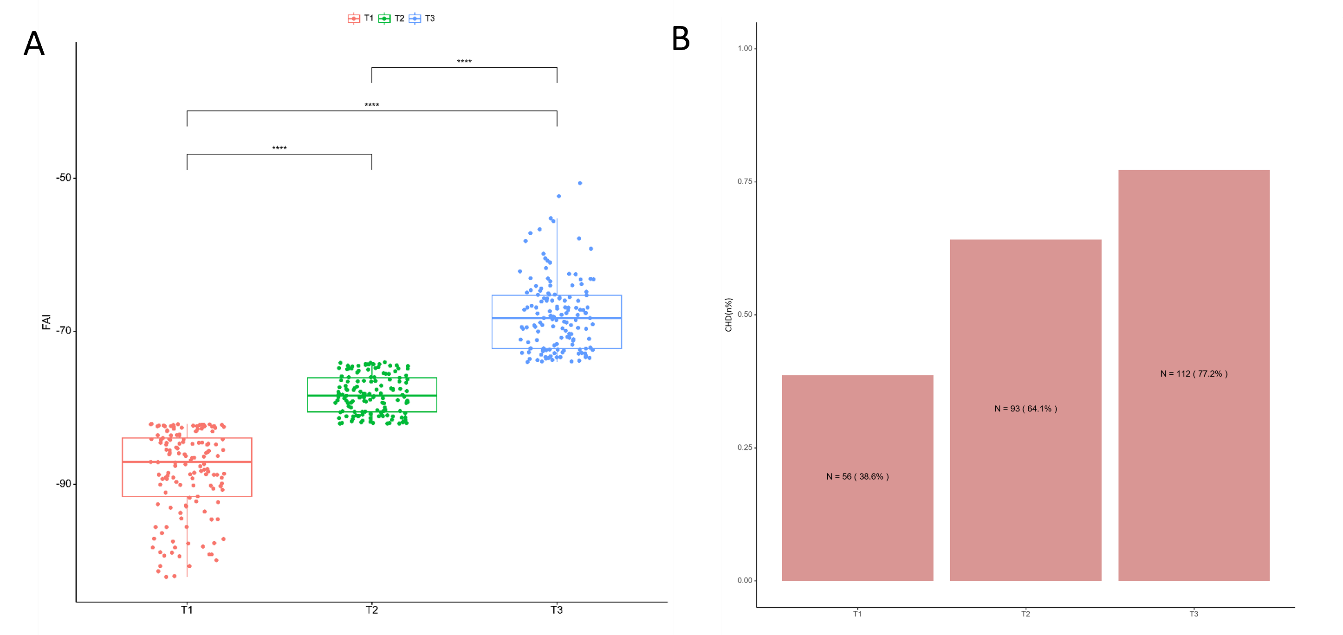


**Figure S1.** Analysis of FAI values and CHD incidence in MAFLD patients across different FAI groups. (A) The distribution of FAI values in T1, T2, and T3 groups and the inter-group differences (box plot). (B) Bar chart comparison of the incidence of CHD in MAFLD patients across T1, T2, and T3 groups.


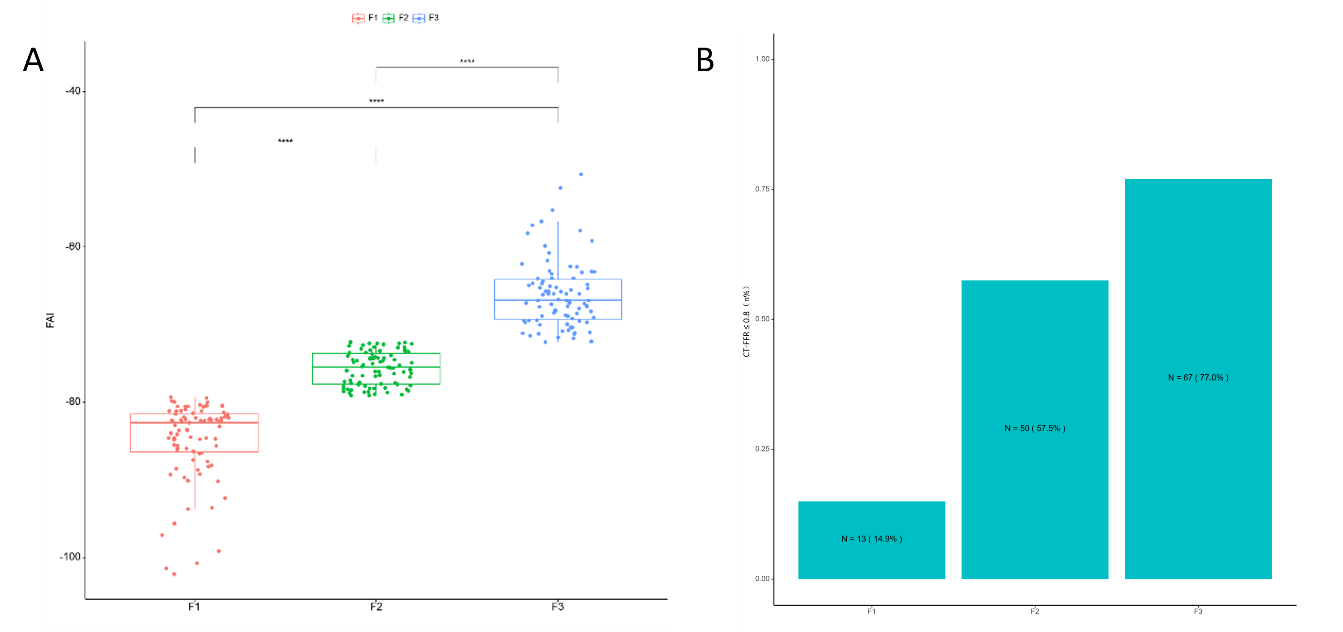


**Figure S2.** Analysis of FAI values and CT-FFR ≤ 0.80 incidence in MAFLD with CHD patients across different FAI groups. (A) The distribution of FAI values and inter-group differences in F1, F2, and F3 groups (box plot). (B) Bar chart comparison of the incidence of CT-FFR ≤ 0.80 in MAFLD patients with CHD across F1, F2, and F3 groups.


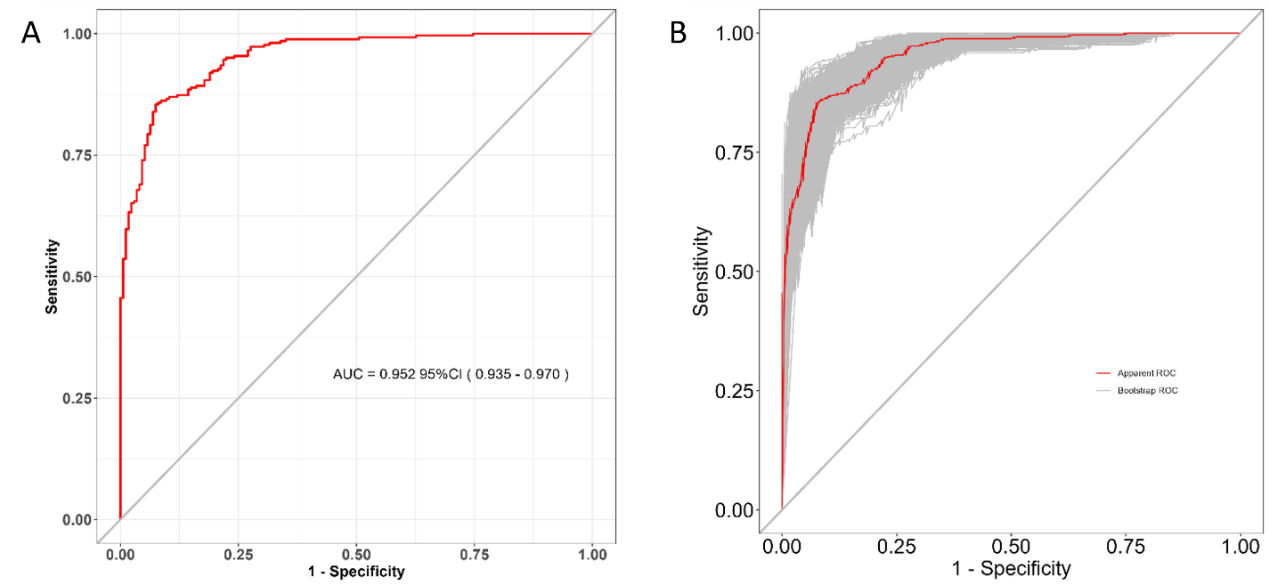


**Figure S3.** ROC Curve and Internal Validation of the Nomogram Model for Predicting CHD in MAFLD Patients (A) ROC curve and AUC of the nomogram model (B) ROC curve from bootstrap internal validation of the nomogram model. The apparent ROC represents the ROC curve based on the original dataset, while the bootstrap ROC was generated from 1,000 resampling iterations for internal validation. The gray shading indicates the 95% confidence interval. Validation results demonstrate that the model has good discriminative ability and stability


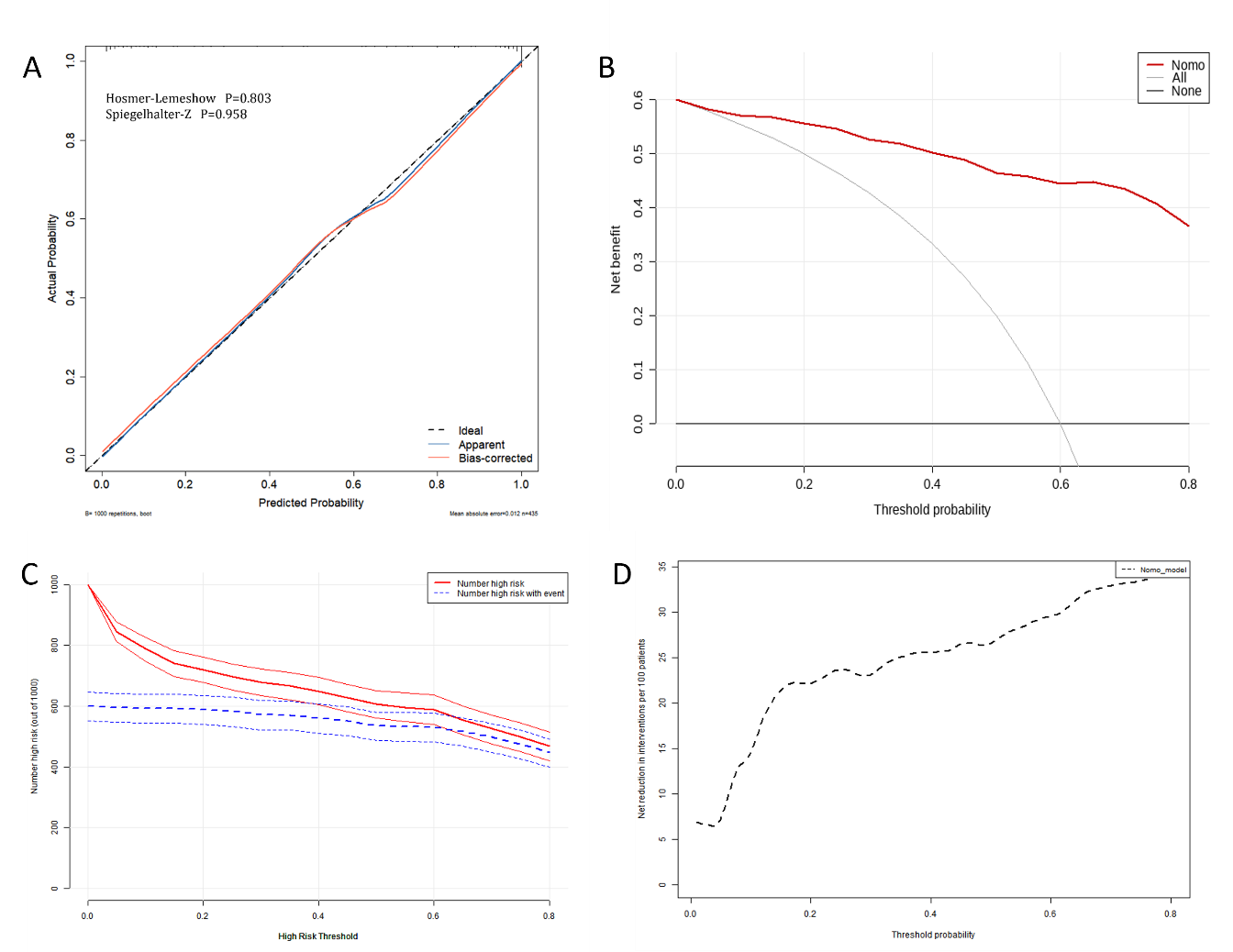


**Figure S4.** Validation of the Predictive Performance and Clinical Utility of the Nomogram Model for CHD Risk in MAFLD Patients (A) Calibration curve of the nomogram model for predicting CHD risk in MAFLD patients (B) Decision curve analysis of the nomogram model (C) Clinical impact curve of the nomogram model (D) Net reduction curve of the nomogram model


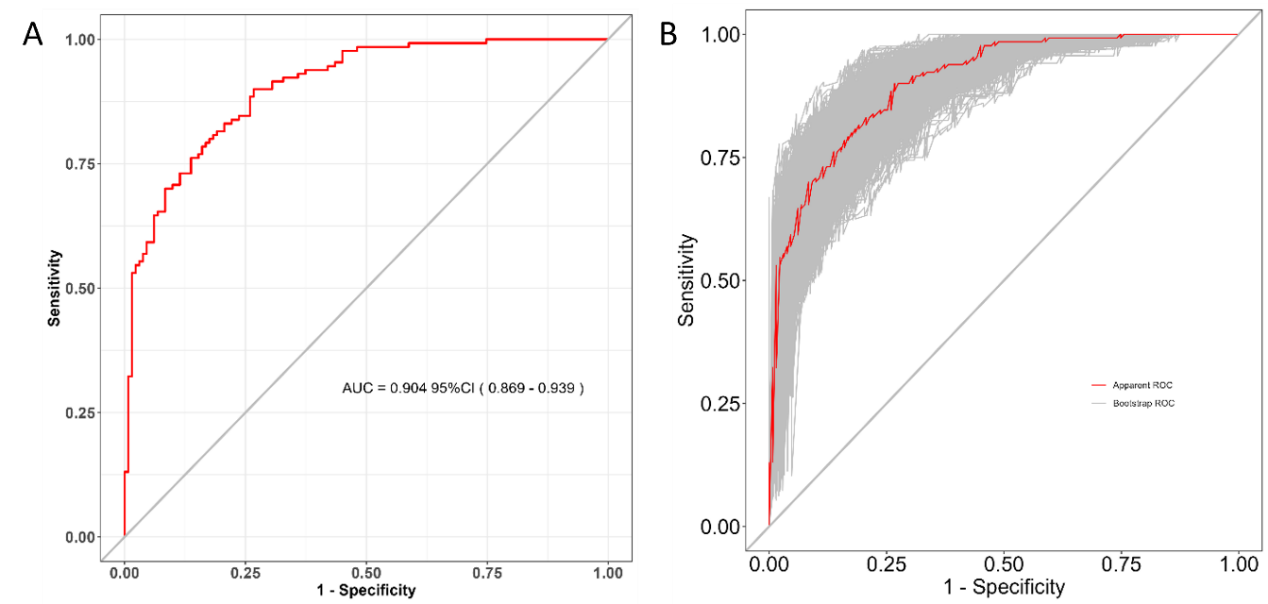


**Figure S5.** ROC Curve and Internal Validation of the Nomogram Model for Predicting Coronary Functional Ischemia in MAFLD Patients with CHD (A) ROC curve and AUC of the nomogram model (B) ROC curve from bootstrap internal validation of the nomogram model. The apparent ROC represents the ROC curve based on the original dataset, while the bootstrap ROC was generated from 1,000 resampling iterations for internal validation. The gray shading indicates the 95% confidence interval. Validation results demonstrate that the model has good discriminative ability and stability


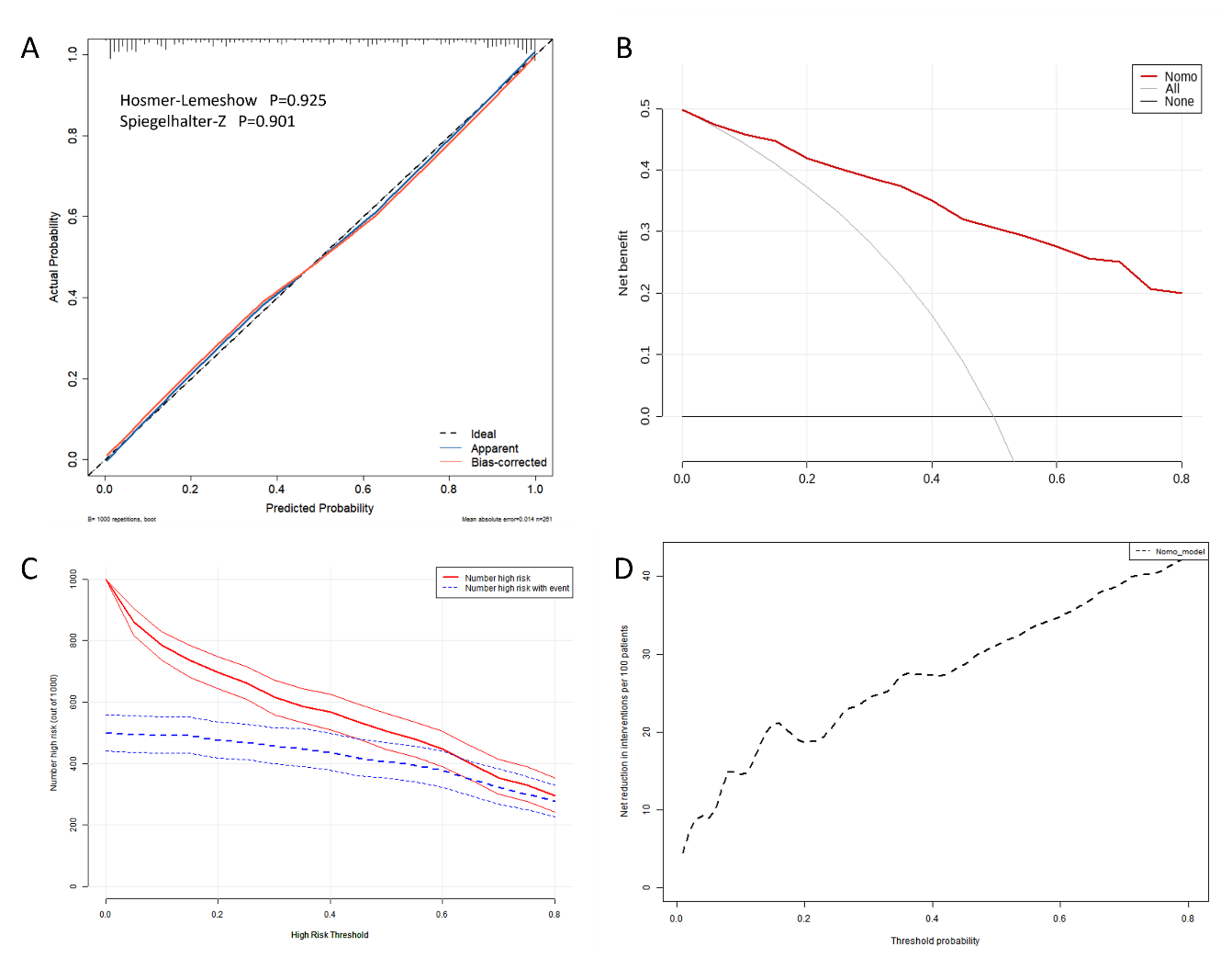


**Figure S6.** Validation of the Predictive Performance and Clinical Utility of the Nomogram Model for Coronary Functional Ischemia Risk in MAFLD Patients with CHD (A) Calibration curve of the nomogram model for predicting coronary functional ischemia risk (B) Decision curve analysis of the nomogram model (C) Clinical impact curve of the nomogram model (D) Net reduction curve of the nomogram model


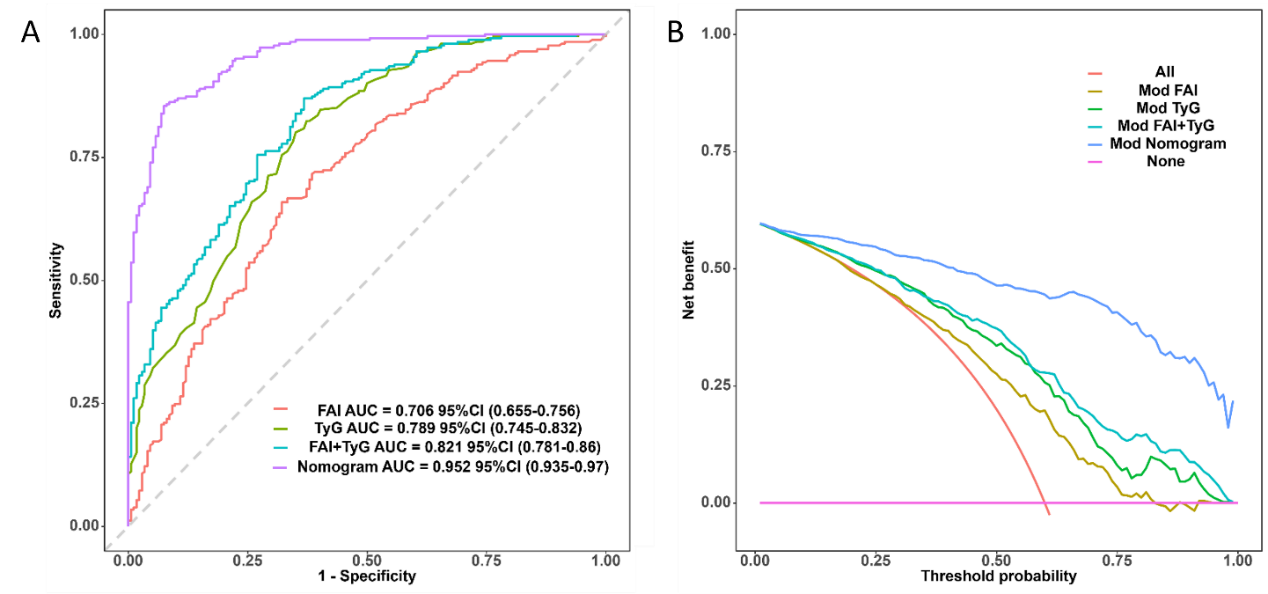


**Figure S7.** Comparative Analysis of the Predictive Performance of the TyG Index, FAI, Their Combination, and the Nomogram Model for CHD Risk in MAFLD Patients (A) ROC curves of the TyG index, FAI, their combination, and the nomogram model for predicting CHD risk in MAFLD patients (B) Decision curve analysis of the TyG index, FAI, their combination, and the nomogram model for predicting CHD risk in MAFLD patients.


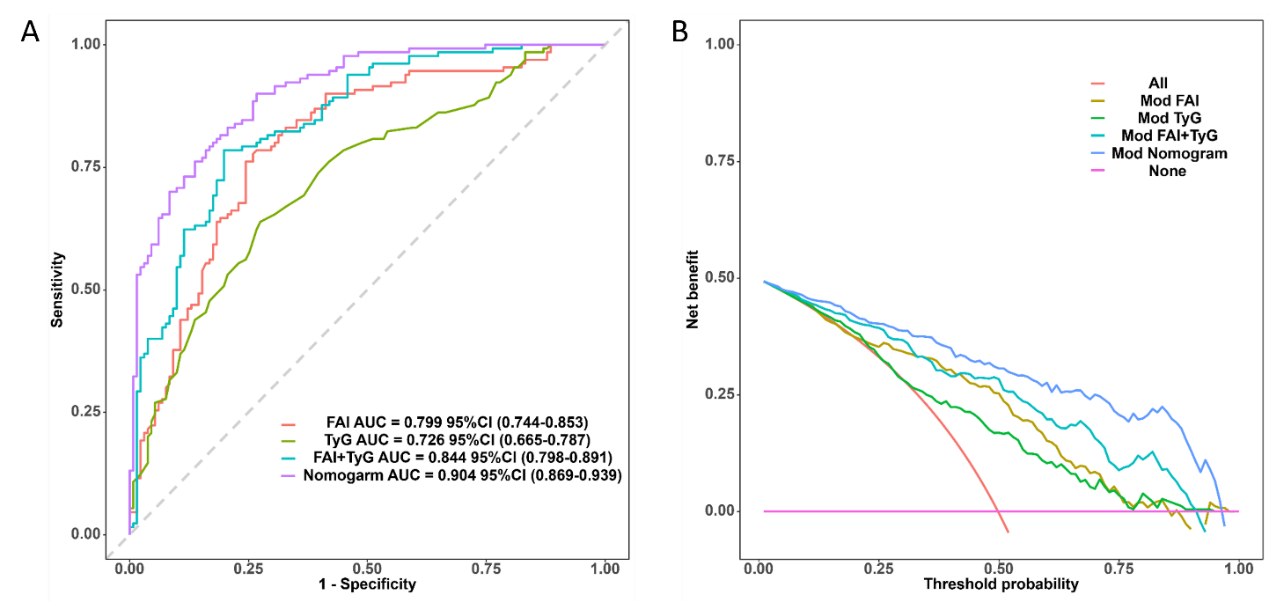


**Figure S8.** Comparative Analysis of the Predictive Performance of the TyG Index, FAI, Their Combination, and the Nomogram Model for Coronary Functional Ischemia Risk in MAFLD Patients with CHD (A) ROC curves of the TyG index, FAI, their combination, and the nomogram model for predicting coronary functional ischemia in MAFLD patients with CHD (B) Decision curve analysis (DCA) of the TyG index, FAI, their combination, and the nomogram model for predicting coronary functional ischemia in MAFLD patients with CHD

**Table S1**. Comparison of baseline data between the MAFLD group and the CHD + MAFLD group.

|  | Total（N=435） | MAFLD（N=174） | CHD＋MAFLD（N=261） | p |
| --- | --- | --- | --- | --- |
| Male（n%） | 265(60.9%) | 83(47.7%) | 182(69.7%) | ***<0.001*** |
| Age (years) | 55.8±9.71 | 53.2±9.73 | 57.5±9.32 | ***<0.001*** |
| Smoking (n%) | 192(44.1%) | 54(31.0%) | 138(52.9%) | ***<0.001*** |
| Diabetes mellitus (n%) | 114(26.2%) | 26(14.9%) | 88(33.7%) | ***<0.001*** |
| Hypertension (n%) | 196(45.1%) | 71(40.8%) | 125(47.9%) | 0.175 |
| BMI(kg/m^2^) | 26.3±3.55 | 25.9±3.83 | 26.5±3.34 | 0.098 |
| WBC(*10^9/L) | 6.86±2.13 | 6.05±1.81 | 7.41±2.16 | ***<0.001*** |
| NEU(*10^9/L) | 4.70±1.66 | 4.30±1.88 | 4.97±1.43 | ***<0.001*** |
| ALT(U/L) | 26.2[23.0;30.6] | 23.2[20.5;26.2] | 28.7[24.9;32.3] | ***<0.001*** |
| AST(U/L) | 22.2[20.0;24.6] | 20.2[18.6;22.1] | 23.4[21.5;25.8] | ***<0.001*** |
| TC(mmol/L) | 4.61±1.19 | 4.56±1.12 | 4.65±1.23 | 0.388 |
| TG(mmol/L) | 1.93[1.75;2.18] | 1.81[1.49;2.12] | 1.99[1.83;2.22] | ***<0.001*** |
| FBG(mmol/L) | 5.88[5.36;6.58] | 5.46[5.17;5.90] | 6.24[5.70;7.01] | ***<0.001*** |
| Scr(mmol/L) | 68.7±12.2 | 67.2±11.5 | 69.7±12.5 | ***0.029*** |
| HDL-C(mmol/L) | 1.19[1.07;1.30] | 1.22[1.11;1.32] | 1.13[1.02;1.28] | ***<0.001*** |
| LDL-C(mmol/L) | 2.51[2.38;2.67] | 2.50[2.37;2.66] | 2.51[2.38;2.68] | 0.513 |
| FAI(HU) | -78.18±9.50 | -82.18±9.28 | -75.52±8.69 | ***<0.001*** |
| TyG | 9.14[8.95;9.29] | 8.96[8.73;9.16] | 9.21[9.10;9.34] | ***<0.001*** |

**Table S2**. Comparison of baseline characteristics between patients with coronary functional ischemia and those without coronary functional ischemia in the CHD + MAFLD group.

|  | Total（N=261） | CT-FFR＞0.80（N=131） | CT-FFR≤0.80（N=130） | p |
| --- | --- | --- | --- | --- |
| Male（n%） | 182(69.7%) | 88(67.2%) | 94(72.3%) | 0.443 |
| Age (years) | 57.5±9.32 | 57.7±9.53 | 57.4±9.13 | 0.809 |
| Smoking (n%) | 138(52.9%) | 65(49.6%) | 73(56.2%) | 0.351 |
| Diabetes mellitus (n%) | 88(33.7%) | 41(31.3%) | 47(36.2%) | 0.485 |
| Hypertension (n%) | 125(47.9%) | 58(44.3%) | 67(51.5%) | 0.293 |
| BMI(kg/m^2^) | 26.5±3.34 | 26.3±3.17 | 26.7±3.50 | 0.416 |
| WBC(*10^9/L) | 7.41±2.16 | 6.81±2.01 | 8.01±2.15 | ***<0.001*** |
| NEU(*10^9/L) | 4.97±1.43 | 4.68±1.41 | 5.26±1.41 | ***<0.001*** |
| ALT(U/L) | 28.7[24.9;32.3] | 28.7[24.4;31.9] | 28.5[25.4;32.6] | 0.393 |
| AST(U/L) | 23.4[21.5;25.8] | 22.9[21.2;25.5] | 23.9[21.6;26.2] | 0.244 |
| TC(mmol/L) | 4.65±1.23 | 4.56±1.20 | 4.75±1.26 | 0.215 |
| TG(mmol/L) | 1.99[1.84;2.22] | 1.97[1.83;2.13] | 2.05[1.85;2.28] | ***0.025*** |
| FBG(mmol/L) | 6.24[5.70;7.01] | 6.00[5.66;6.52] | 6.78[6.01;7.37] | ***<0.001*** |
| Scr(mmol/L) | 69.7±12.5 | 69.1±12.7 | 70.4±12.4 | 0.385 |
| HDL-C(mmol/L) | 1.13[1.02;1.28] | 1.22[1.06;1.36] | 1.09[0.98;1.22] | ***<0.001*** |
| LDL-C(mmol/L) | 2.51[2.38;2.68] | 2.50[2.36;2.67] | 2.52[2.41;2.69] | 0.298 |
| FAI(HU) | -75.52±8.69 | -79.77±7.98 | -71.23±7.16 | ***<0.001*** |
| TyG | 9.21±0.19 | 9.14±0.18 | 9.29±0.17 | ***<0.001*** |
